# The Interaction of Diet and Mitochondrial Dysfunction in Aging and Cognition

**DOI:** 10.3390/ijms22073574

**Published:** 2021-03-30

**Authors:** Aleksandra Kaliszewska, Joseph Allison, Matteo Martini, Natalia Arias

**Affiliations:** 1 Department of Basic and Clinical Neuroscience, Institute of Psychiatry, Psychology and Neuroscience, King’s College London, Denmark Hill, London SE5 8AF, UK; aleksandra.kaliszewska@kcl.ac.uk (A.K.); joseph.c.allison@kcl.ac.uk (J.A.); 2 Department of Psychology, University of East London, London E154LZ, UK; m.martini@uel.ac.uk; 3 Instituto de Neurociencias del Principado de Asturias (INEUROPA), 33005 Oviedo, Spain

**Keywords:** aging, cognitive impairment, diet, neuroinflammation, stress

## Abstract

Aging is inevitable and it is one of the major contributors to cognitive decline. However, the mechanisms underlying age-related cognitive decline are still the object of extensive research. At the biological level, it is unknown how the aging brain is subjected to progressive oxidative stress and neuroinflammation which determine, among others, mitochondrial dysfunction. The link between mitochondrial dysfunction and cognitive impairment is becoming ever more clear by the presence of significant neurological disturbances in human mitochondrial diseases. Possibly, the most important lifestyle factor determining mitochondrial functioning is nutrition. Therefore, with the present work, we review the latest findings disclosing a link between nutrition, mitochondrial functioning and cognition, and pave new ways to counteract cognitive decline in late adulthood through diet.

## 1. Introduction

Cognition, broadly defined as encompassing the mental processes of learning, reasoning and memory, depends on the brain’s ability to undergo functional (e.g., adjusting efficacy of synaptic transmission) and structural (e.g., forming new synapses) alterations in response to changes in its environment—a phenomenon known as neuroplasticity. Internal and external stimuli—such as new experiences and learning, trigger changes in neuronal activity which induce re-organisation of neuronal networks and fine-tuning of brain circuitry, with new connections between neurons made that can be strengthened, or weakened or pruned away. The neurobiological processes underpinning both cognitive development and functioning such as neurogenesis, synaptic remodelling and neurotransmission, are energetically costly and rely on an uninterrupted supply of mitochondrial adenosine triphosphate (ATP) [1].

With an average human cortical neuron consuming 4.7 billion ATP molecules per second for ‘housekeeping’ activities and maintenance of its membrane potential and ion homeostasis, mitochondria must produce a staggering 5.7 kg of ATPs each day just to support basic brain function (that’s 5 times the brain’s own weight!) [2]. Therefore, even though the brain accounts for just 2% of the average human body mass, it’s the most energetically taxing, utilising 25% of total energy supplies [3]. Moreover, 70–80% of that energy is used by neurons [2] and primarily at synapses, where mitochondria are largely localized and needed to support the energetic expenditure associated with information processing and propagation of electrical signals [4].

While the unproportionally high energy requirement of neurons has almost certainly played a central role in the development of human’s uniquely superior cognitive abilities, it has also rendered the brain highly vulnerable to perturbances in energy supply. Not surprisingly, mitochondrial disorders, characterised by bioenergetic failure resulting from mutations in nuclear and/or mitochondrial genes, predominantly affect the brain and commonly cause cognitive impairments ranging from mild to severely debilitating [5]. Even physiologically healthy aging is associated with reduced availability of glucose for mitochondrial phosphorylation, diminished activity of electron transport chain, deficient antioxidant capacity, and breakdown of mitochondrial energetic function. All these processes are marked by morphological disturbances and alterations in genes regulating mitochondrial biogenesis, which correlate with cognitive decline occurring in later life.

**Figure 1:**
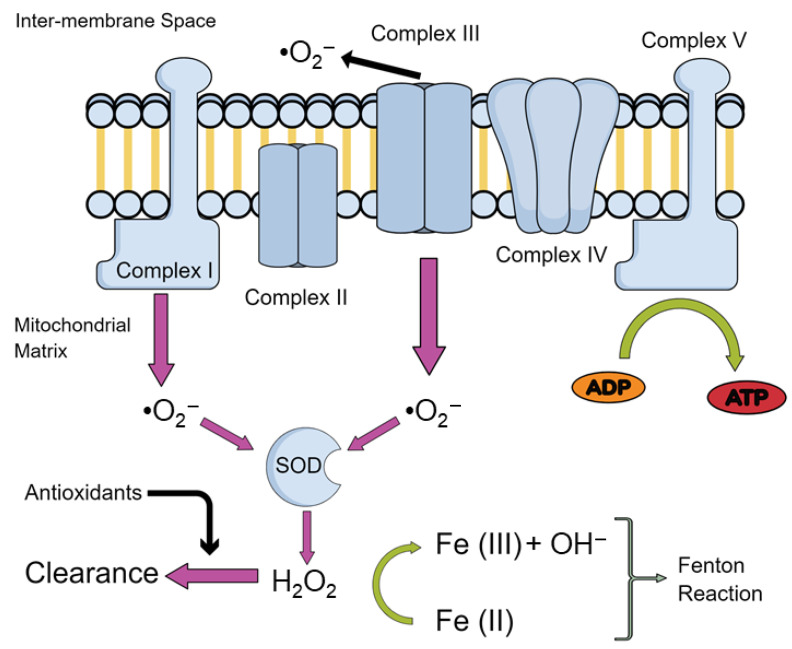
Production of ROS in Mitochondria. An overview of the production of the main reactive oxygen species (ROS) in the mitochondria. Abbreviations: Superoxide ions, •O_2_^−^; iron ions, Fe (II)/(III); adenosine diphosphate, ADP; adenosine triphosphate, ATP; superoxide dismutase, SOD; hydrogen peroxide, H_2_O_2_.

Therefore, the study of mitochondrial impairments is of growing interest in order to unravel the mechanism leading to normal ageing. One of the more prominent theories revolves around the production of reactive oxygen species (ROS). The mitochondrial theory of ageing postulates that production of these ROS derive mainly from oxidative phosphorylation (OXPHOS) taking place within mitochondria. An inevitable by-product of electron transport chain (ETC) activity, responsible for OXPHOS, is the formation of superoxide anion radicals (O_2_^−^), mostly by complexes I and III [6] and hydroxyl (OH^−^) via iron-mediated reduction, known as the Fenton reaction [7]—overviewed in Figure 1.

Under physiological conditions, ROS are involved in processes such as immune response, inflammation, as well as synaptic plasticity, learning, and memory [8,9]. However, when produced in excess, ROS production may overwhelm antioxidant defences, leading to impairments of cellular function such as damaging proteins and deoxyribonucleic acid (DNA), and inducing lipid peroxidation which leads to mitochondrial dysfunction observed in aging [10].

Diet has emerged as a critical component underlying aging-related cognitive deficits. It has been proven that unhealthy diets have an effect on cognition through the induction of an inflammatory response in the aged brain [11,12,13,14]. Given the prevalence of neuroinflammation in memory impairments, highly prevalent in aged individuals, lifestyle modifications with special emphasis on diet present a promising potential intervention [15,16,17]. Indeed, adherence to the Mediterranean diet [18] has been associated with better cognitive function [19,20] and slower cognitive decline [19,20,21], though some studies have reported null findings [22,23,24].

In this review, we will discuss the role of mitochondrial dysfunction in the aging process, with a specific focus on cognitive deterioration. We will also give some insights into the dual role diet plays in mitochondrial dysfunction while aging, and the dietary strategies to better understand the age-related mitochondrial impairments.

## 2. ROS and Its Effects on the Mitochondria

As we have previously described, the imbalance between free radical production and detoxification leads to bioenergetic impairments as well as disturbances in the reduction-oxidation (redox) homeostasis in the brain with increasing age. Indeed, post-mortem brain samples from 80-year-old subjects have revealed a progressive age-related rise in protein nitration and oxidation, together with a decrease in antioxidant defences such as superoxidase dismutase (SOD), catalase (Cat), and glutathione (GSH) reductase activity, as well as mitochondrial complex I activity, mainly in the hippocampus and frontal cortex [25]. Moreover, Mandal et al. (2012) [26] have found a gradual decrease in GSH content in the human brain of 56-years-old subjects. More interestingly, this age-related increase in brain oxidative stress was even greater in individuals with high body mass index and in smokers, highlighting the influence of lifestyle in the processes of aging. In line with these findings, Rebrin et al. (2007) [27] found a decrease in the reduced/oxidized glutathione (GSH/GSSG) ratio in the cortex, striatum and the hippocampus of mice, which are brain regions linked to age-related loss of cognitive function.

During aging, a mitochondrial depolarization process and uncoupling of the OXPHOS have been found due to the aperture of the mitochondrial permeability transition pore (mPTP) [28]. The mPTP allows free movement of molecules into the mitochondria that weigh less than 1.5 kilo-Dalton. Whilst under healthy conditions this is hypothesised to act as an immediate resource dump of ions such as calcium, prolonged opening is associated with pathological conditions [29]. ROS may activate the mPTP, which results in the activation of an apoptotic pathway via the release of cytochrome c oxidase and initiation of the caspase-9 cascade [30]. mPTP activation may also result in further production of ROS as well as in the activation of the nod-like receptor pyrin domain 3 (NLRP3) inflammasome [31]. This latter finding is relevant since the activation of NLRP3 in microglia and astrocytes in the brain are crucial for neuronal loss in nigrostriatal neurons and remains a key mechanism through which motor deficits and cognitive deficits are manifested in Parkinson’s disease and other disorders [31,32,33]. Moreover, it has been shown that the mPTP is also more susceptible to activation in older mice, thus resulting in increased apoptosis in aged animals [34]. Furthermore, tumour necrosis factor alpha (TNF-α) has been reported to cause aberrant opening of mPTP, suggesting that agents which increase inflammation with subsequent release of TNF-α, such as high fat diets [35,36], may also result in an increase in mPTP. These results suggest that mitochondrial dysfunction, ROS, and inflammatory by-products are responsible for age-related cognitive deterioration and can be further modulated by dietary changes.

### 2.1. Oxidative Damage to Lipids and Protein in Aging

Aging has been shown to increase the oxidative damage to DNA, lipids [37,38,39] and proteins [40,41]. Among phospholipids, cardiolipin became relevant not only for being almost exclusively located in the inner mitochondrial membrane, where it is biosynthesized [42,43,44], but also because evidence suggested that cardiolipin is involved in the regulation of key mitochondrial inner membrane proteins’ activity involved in oxidative phosphorylation [38,39,45]. Additionally, due to its location near the site of ROS production, cardiolipin is particularly prone to be peroxidised. Indeed, oxidative stress readily decreases cardiolipin, which results in a decrease in cytochrome c oxidase, a key enzyme in the ETC [46,47]. These results point out that the oxidation/depletion of cardiolipin with aging contribute to mitochondrial dysfunction, leading to cellular dysfunction and eventually cell death.

Lipid peroxides may themselves have a pathogenic role by attacking amino acid residues in proteins such as cytochrome c oxidase, ATPase, and nicotinamide adenine dinucleotide hydrogen (NADH) dehydrogenase, that are prone to oxidation [48,49,50,51,52]. This results in heterogenous chemical bonding linking both the protein and lipid together causing peroxidative damage due to loss of solubility [53]. Peroxidative damage can reduce mitochondrial cytochrome c oxidase activity, but may have more widespread implications through peroxidation of synaptic proteins, which impairs cognition [54,55,56]. Protein oxidation may also occur due to direct interaction with ROS through redox reactions. Additionally, superoxide ions can cause iron-sulphur centres in complex I of the ETC to expel iron, thereby increasing the concentration of labile iron molecules [57]. Iron is necessary for the reduction of hydrogen peroxide to hydroxide ions via the Fenton reaction [7], with an increase in iron concentration being linked to ROS production as well as cellular death and aging [58].

### 2.2. Lipofuscinosis as a Mitochondrial Dysfunction Marker in Aging

Interestingly, iron accumulates in a heterogenous aggregate known as lipofuscin. Lipofuscin is known as the wear and tear pigment, containing indigestible proteins, lipids and metals, and positively correlates with aging and oxidative stress [59,60,61,62,63,64,65,66,67]. Despite being a well described feature of aging, the exact mechanism through which these macromolecules accumulate remains unknown, as well as any function or deleterious effect they may pose to the cell [64]. For example, increased intracellular concentrations of iron increase ROS, but it is not known whether sequestered iron in lipofuscin is catalytically active in forming hydroxide ions. Lipofuscin may act as a protective accumulate in this instance by removing the cell’s potential to create ROS. Moreover, a large constituent of lipofuscin is a subunit c of mitochondrial ATP Synthase (SCMAS), the final complex in the ETC [68]. Why this particular subunit accumulates remains unexplored, however, it is hypothesised that SCMAS may be required in the formation of the mPTP. Therefore, perhaps accumulations of SCMAS reflect an equal increase in mPTP. This might indicate early mitochondrial dysfunction in diseases such as the neuronal ceroid lipofuscinoses as well as lysosomal disruption [69,70]. Moreover, homologs in *C. elegans* have suggested that removal of this protein reduces mitochondrial function as well as reducing lipofuscin aggregates. Despite the reduction in mitochondrial function, lifespan was extended which may suggest that mPTP activation and cell death is increased when SCMAS is present. Alternatively, SCMAS and lipofuscin aggregates may have a different pathogenic role, distinct from the mPTP, in cell death that remains unexplored [71]. Therefore, the interplay between appropriate SCMAS levels to enable proper mitochondrial function and keeping lipofuscin to a minimum may be key to delaying mitochondrial dysfunction in aging.

### 2.3. Oxidative Damage to DNA in Aging

Particularly relevant to aging is the oxidative damage to DNA, specifically mitochondrial DNA (mtDNA) which is localised close to where the production of ROS occurs. Damage to mtDNA persists longer and repairs less easily than nuclear DNA, making it particularly vulnerable to perturbation [72]. mtDNA only contains one non-coding region and therefore makes mutations in exonic regions very high—likely to give rise to frameshift mutations or deletions. Lesions to mitochondrial DNA such 7,8-dihydro-8-oxo-deoxyguanosine (8-oxo-dG) caused by ROS are indicative of the extent of oxidative stress, and its presence has been noted in mtDNA of aged animals [73,74,75,76]. As a consequence, DNA repair mechanisms to 8-oxo-dG lesions via mitochondrial apurinic/apyridinimic endonucleases are upregulated in aged rats compared to younger rats [77]. Whilst the same study reported an increase in the DNA glycosylase repair mechanism, another study has shown reductions of DNA glycosylase in old mice, making it difficult to draw conclusions [78]. Indeed, the vast majority of DNA repair mechanisms reduce in activity with age, so it is possible that is a very specific increase to 8-oxo-dG lesions [79,80].

Accumulation of mtDNA mutations via ROS has been suggested as a central mechanism driving aging and age-related diseases [81,82,83,84,85,86] due to their contribution to cellular senescence [87], a process which halts cell division and thereby prevents new cell formation. The cause of senescence is believed to be the shortening of telomeres, which are the caps that protect chromosomes from damage response enzymes [88]. Telomere shortening is directly related to oxidative stress levels with ROS, able to manipulate telomere maintenance through multiple pathways [89,90]. ROS-induced 8-oxo-dG lesions in mtDNA have been shown to reduce the ability for telomerase to bind to telomeres as well as reduce the activity of the reverse transcriptase subunit of telomerase [91,92]. Telomerase is critical for the extension of telomere length due to its ability to add guanine-rich repeats and increase the length of the chromosome. Antioxidants have been shown to increase the activity of the reverse transcriptase subunit and delay cellular senescence [92]. Furthermore, female rats fed a telomerase activator-65 (TA-65, which increases telomerase activity), purified from the root of Chinese herbs such as Astragalus membranaceus, after a brain injury showed increased cognitive function, movement, and reductions in depressive-like behaviours [93]. Other studies support these findings and also show telomerase-dependent neurogenesis and an increased lifespan, as well as the ability to supplement TA-65 through diet [94] or increase telomerase directly through activities such as meditation [95], which may be able to delay the onset of cellular senescence [94,96].

In addition to telomerase binding, 8-oxo-dG also reduces TTAGGG repeat binding factor 1 (TRF1) and 2 (TRF2) binding affinities. These proteins are components of the telomere cap, thus the presence of mtDNA lesions renders chromosomes more vulnerable to DNA damage response enzymes [97,98,99]. ROS builds upon this vulnerability by also upregulating the DNA-damage response, which increases levels of p53 [100,101]. P53 then positively feeds back to regulate telomeric capping by ubiquitination and degradation of TRF2, thereby destabilizing the telomere caps and causing early onset senescence [102,103].

## 3. Mitochondrial Dysfunction and “Inflamm-Aging”

Mitochondria are highly adapted organelles when maintaining homogeneity within the cellular population. Through fusion and fission, mitochondria can dilute their contents, such as mtDNA, metabolites, and proteins, as well as quality control and redistribute to other areas of the cell [104]. Highly damaged mitochondria from ROS present a risk to the cell, and so removal mechanisms such as mitophagy are critical. Mitophagy avoids the accumulation of such mutations through two opposing mechanisms, fission and fusion. Fission enables the renewal, redistribution, and proliferation of mitochondria, whereas fusion allows them to interact and communicate with each other and facilitates mitochondrial movement and distribution across long distances [105]. Fusion events are particularly important for the enrichment of mtDNA via dilution of mutations. It has been shown that dynamin-related protein 1 (Drp1) ablation, which is a conserved dynamin-related guanosine triphosphate hydrolase (GTPase) involved in fission processes, caused alterations in mitochondrial morphology, such as sphericity and general size, reductions in neuritic mitochondria via translocation away from presynaptic terminals, and both a decrease in oxygen consumption and ATP production [106]. On a broader scale, the loss of Drp1 affected synaptic transmission by reduced ability to generate action potentials under high-stimulus load as well as impairing short-term hippocampal-dependent memory function, demonstrating the critical role of mitochondrial fusion/fission activity in brain function. Furthermore, in studying age-related cochlea degeneration, Drp-1 was decreased in aging and senescent cells as well as mitophagy, potentially suggesting that some age-related cognitive deficits are a result of Drp-1 insufficiency [106,107].

### Inflamm-Aging: Inflammatory-Mitochondrial Dysfunction in Aging

Whilst mitochondria may undergo fusion and fission, another alternative is to release harmful molecules such as oxidised mtDNA, proteins, and cardiolipin into extracellular vesicles (EV) which can then be degraded (reviewed in [108]).

Stimulation of EV formation can occur due to oxidative stress via oxidation of cardiolipin and curvature of the mitochondrial membrane, PTEN-induced kinase 1 (PINK1) accumulation, and subsequent Parkin recruitment. Through mediation of, as of yet, unknown proteins, the EVs are formed following Parkin recruitment [109]. It is proposed that mtDNA, ROS, cardiolipin, and other constituents of these EVs act as DAMPs, damage associated molecular patterns and initiate inflammatory responses which can contribute to aging [110,111,112,113,114]. Moreover, EV formation increases with age and this, along with mtDNA release, correlates with release of pro-inflammatory cytokines [115].

Both ROS and cardiolipin can incite inflammation through Toll-Like Receptors by the activation of nuclear factor kappa B (NF-kB), which activates the pro-inflammatory cytokines tumour necrotic factor alpha (TNF-a), as well as activating T-lymphocytes in an immune response through the interleukin-2 cluster of differentiation (CD28)-dependent pathway [116,117]. With ROS and cardiolipin’s effects increasing with age they may increase the total amount of activated T cells, which may explain the presence of increased T cell infiltration in the aging brain [118,119,120,121].

Moreover, mtDNA acts through the NLRP3 inflammasome with oxidized variants of mtDNA serving as the ligand, indicating that ROS-induced 8-oxo-dG lesions may be a critical component of inflammation in aging [122,123]. Indeed, mitochondrial dysfunction and the presence of cardiolipin is essential for activation of NLRP3 [112]. Downstream consequences of NLRP3 induction include caspase-1 activation which cleaves Parkin preventing mitophagy [124]. With fission and fusion no longer achievable, oxidized macromolecules build up causing pyroptosis—inflammation mediated cell death—via activation of interleukin-1β (IL-1β) and interleukin-18 [125]. Following cell death, the contents of the cell leak with mtDNA and ROS are free to interact with additional inflammasomes and exaggerate the inflammatory response further [123].

## 4. Mitochondria Dysfunction and Immunity in Aging

Perturbations in mitochondria may result in decreased biosynthesis of energy. In T-cells, there is high metabolic demand for extensive proliferation and genetic remodelling [126]. Inefficient mitochondria may therefore fail to reach the required energy levels. This may be particularly key for T memory cells, which rely on the ETC, but must rapidly activate upon pathogenic invasion. Mitochondrial dysfunction therefore may explain the decrease in efficacious prophylaxis in the elderly [127,128,129,130]. Moreover, cytochrome c oxidase-a complex in the ETC-is crucial in T cell proliferation with a 50% reduction in mice that contain mutations in cytochrome c oxidase. This resulted in a 1.6 fold increase of dead cells and 2.5 fold increase in dying cells [131]. Therefore, any alterations in mtDNA encoding cytochrome c oxidase or direct protein oxidation of the enzyme may result in the loss of T cell function and early T cell death, which might explain why the elderly are immunocompromised [127,131]. Moreover, mitochondrial dysfunction has been linked to immune-senescence of T-cells [132] and microglia in the brain [133]. This may result in abnormal activation towards harmful stimuli. Both T-cells and microglia display the propensity to both promote inflammation and attenuate it through different pathways, however, in microglia, mitochondrial dysfunction impairs the ability to adopt a neuroprotective role and may therefore push microglia towards a pro-inflammatory response [134,135].

It has been suggested that microglia’s neuroinflammatory processes in aging lead to downstream changes that damage the brain’s synaptic plasticity functions, ensuing in memory impairment. Moreover, microglial activation promotes subsequent astrocytic activation as illustrated by Clarke et al. (2018) who demonstrate that mice lacking microglial-secreted cytokines had reductions in reactive astrocyte genes which are normally upregulated in aging [136]. Therefore, astrocytes also play a central role in neuroinflammation and brain aging [137,138,139]. More importantly, it has been proven that diets are able to induce a robust inflammatory response in the aged brain, such as high-fat content diets, which not only induce memory deficits in aged rodents, but also increase pro-inflammatory cytokines and reduce phagocytic activity of microglia [140,141].

**Figure 2:**
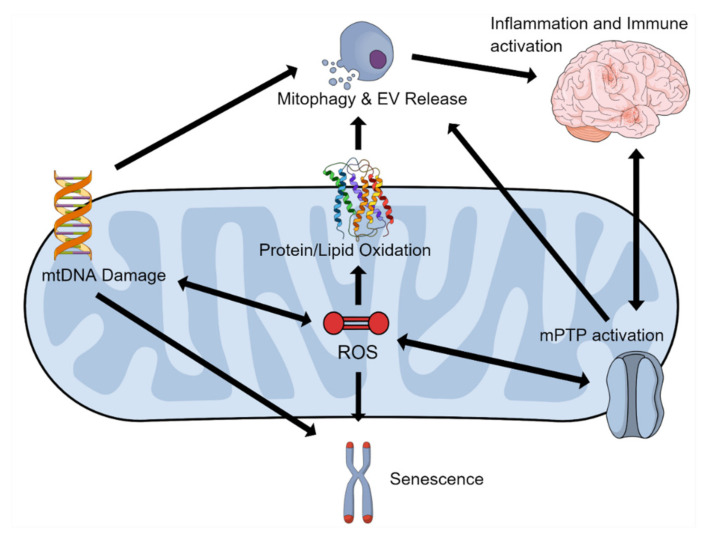
The Effects of ROS on Mitochondrial Function. An overview of the effects that generation of reactive oxygen species (ROS), classically associated with aging, have on mitochondrial function and DNA (mtDNA). Abbreviations: Extracellular vesicles, EV; mitochondrial permeability transition pore, mPTP.

With the mitochondria being involved in so many pathological hallmarks of aging (overviewed in Figure 2), there is a very strong case for the mitochondrial theory of aging. As such, these organelles remain a key focal point for age-related disorders and disruptions by increasing ROS and by inducing DNA mutations. As such, mitochondria position themselves as a key therapeutic target acting as the major confluence for many age-related disorders and deleterious aging effects.

## 5. Mitochondria Dysfunction and Cognitive Impairment in Aging

One of the hallmarks of brain aging, alongside cortical atrophy [142], synaptic loss [143] and low-grade chronic inflammation [144], is cerebrovascular pathology. Alterations in macro and microvasculature disrupt the integrity of blood vessels, resulting in diminished cerebral blood flow (CBF), also referred to as hypoperfusion. It has been estimated that CBF decreases by 5% with every decade of life [145], with data from imaging studies providing evidence for reduced cerebral perfusion occurring as part of normal, healthy aging [146,147,148,149]. Age-related hypoperfusion has important implications for mitochondrial energy metabolism and cognitive functions as, despite high energetic demands of neurons, the brain’s intracellular glycogen stores are particularly limited [150]. Such limitation in glycogen availability makes the brain critically reliant on undisrupted CBF for supply of oxygen and glucose—the main energy substrate of mitochondrial OXPHOS. Not surprisingly, reduced cerebral perfusion has been associated with hypometabolisms and worsened cognitive performance in aged individuals, with cerebrovascular dysfunction a commonly reported co-morbidity in dementia [151,152,153,154,155]. Strikingly, even in otherwise healthy individuals, hypertension or persistently high blood pressure is known to impair cognitive function, with potentially severe memory loss occurring in advanced age [156]. It is also important to mention that part of the relationship between hypertension and memory loss may relate to common cause, since factors like oxidative stress and cell energy deficit drive both hypertension and memory loss.

On the other hand, reduced CBF resulting in inadequate delivery of oxygen to cells in the brain induces a state of hypoxic stress, disrupting the permeability of the blood–brain barrier (BBB) [157,158,159]. Breakdown of BBB’s integrity triggers a neuroinflammatory response via upregulation of oxygen-sensitive hypoxia-inducible-factors (HIFs) transcription factors [160], which are implicated in neurodegenerative processes [161]. Hypoxia-induced brain damage is further propagated by neuroinflammatory changes in microglia and astrocytes, which undergo morphological and functional alterations known as reactive gliosis, and which are hypothesized to exacerbate cognitive impairments associated with oxygen depletion in the brain [162,163,164,165]. Following an ischemic insult, severe memory loss, anterograde amnesia, and behavioural disturbances are known to occur in humans [166,167,168], with similar hypoxia-induced impairments in behaviour and cognition replicated using animal models and associated with increase in inflammatory markers and immune cell infiltration due to hypoxia-induced BBB leakage [162,169,170,171,172]. Notably, hypoxia inducible factors (HIFs) are also key regulators of metabolic adaptations to hypoxia. HIF-signalling affects mitochondrial function, determining mitochondrial mass, composition of the ETC, and ultimately the efficacy of mitochondrial energy production [173]. However, the precise molecular mechanisms linking neuroinflammation, mitochondrial hypometabolism, and cognitive dysfunction resulting from acute or age-related chronic ischemia remain to be elucidated.

### 5.1. Glucose Metabolism and Cognition in Aging

The internal environment of the brain is controlled by the blood–brain barrier, which selectively regulates the passage of nutrients and chemicals into the brain and prevents the entry of pathogens and toxins from systemic circulation. Thus, despite being a primary energy substrate, glucose must be actively transported into the brain via glucose transporters (GLUTs) [174]. Deficits in glucose availability and abnormalities in glucose receptors have been associated with aging, with animal models and human data supporting the role of disrupted brain uptake of glucose in cognitive dysfunction. For example, imaging brain neurons in Drosophila using genetically encoded ATP biosensors, has shown age-dependent reduction of ATP concentration in head extracts of aged flies compared to young flies. The reduction in ATP was accompanied by reduced glucose concentration and downregulation of glucose transporter expression [175]. Conversely, dynamic micro positron emission tomography (PET) imaging of aged Fischer 344 rats has demonstrated a significant reduction in glucose uptake as well as a downregulation of insulin-sensing neuronal glucose transporter GLUT3/4 and GLUT1, which mediates transport of glucose across the vascular endothelium [176]. In human subjects, converging evidence from MRI and fluorodeoxyglucose (FDG)-PET imaging data has shown an age-related decline in glucose uptake, correlating with structural and functional changes in various brain regions associated with cognition [177,178,179,180]. Moreover, lowered glucose uptake is considered a predictive marker of progression to mild cognitive impairment and/or Alzheimer’s disease, as cognitively healthy individuals with reduced brain glucose uptake are at higher risk of dementias and cognitive impairments [181,182]. Interestingly, energy deficits associated with aging in Drosophila were linked to disruption of glycolysis [175]—the metabolic pathway upstream of mitochondrial oxidative phosphorylation which breaks down glucose into pyruvate, and which has been shown to become dysregulated with age in healthy human brains [183]. However, while increasing neuronal glucose uptake by overexpression of hGlut3 ameliorated age-related energy deficits in aged flies by restoring youth-like levels of ATP, it did not rescue mitochondrial dysfunction which was present in the neurons of aged flies [175]. While this finding could suggest that enhanced glycolysis independent of mitochondrial health is sufficient to attenuating age-related energetic deficits, it is important to note that the quality of mitochondria in this work was assessed based on ultrastructural imaging alone, without further analysis of any putative age-related alterations in mitochondrial function, and thus warrants further investigation. Perhaps the most significant finding reported in this study however, was that coupling overexpression of hGlut3 with dietary restriction in aged flies, gave rise to more prominent beneficial effects of longevity and locomotor performance [175]. The synergistic effects of hGlut3 overexpression and cornmeal-free diet containing 1% (*w*/*v*) of yeast and glucose in aged flies, were mediated by upregulation of the efficiency of glucose metabolism [175]. Thus, emphasizing the importance of dietary interventions in regulation of energy metabolism in aging.

**Figure 3:**
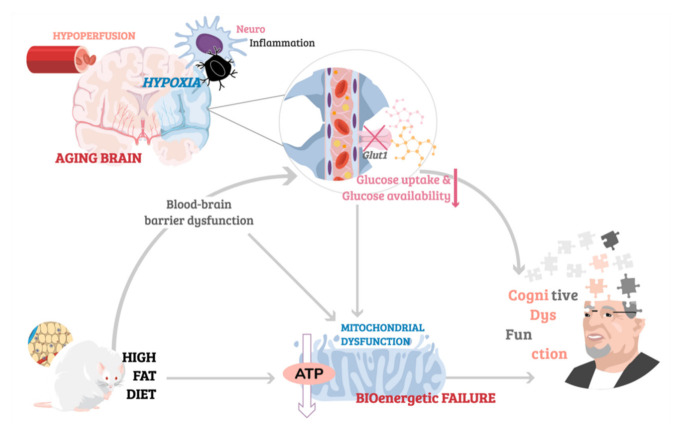
The aging brain, mitochondrial bioenergetic failure and cognition. The link between age-related cerebrovascular dysfunction associated with hypoperfusion and neuroinflammation, nutrition-induced dysregulation of blood-brain barrier permeability, breakdown of glucose metabolism and mitochondrial dysfunction implicated in cognitive impairment in later life.

Conversely, dietary habits associated with high fat content (HFC), are known to impair glucose tolerance and result in insulin resistance, with a plethora of animal studies validating the HFC feeding paradigm as a model of metabolic disturbance [184]. Interestingly, HFC has also been linked to disruption of BBB integrity by inducing changes in BBB permeability reported as increased leakage of dyes such as Evans blue in the brain and decreased expression of tight junction proteins like claudin-5 and occludin [185,186,187]. Moreover, not only does HFC feeding decrease expression of BBB transporter Glut1 [188,189], thus reducing the uptake of glucose, but it has also been shown to induce changes in mitochondrial energy metabolism [189], with proteomics analysis reporting a significant decrease in levels of proteins involved in the ETC in the hippocampus and cerebral cortex of mice fed HFC for two weeks [186]. However, the relationship between HFC and energy metabolism is still poorly understood with many discrepancies in the reported results believed to be due to differences in the duration of HFC feeding (with compensatory mechanisms reported to occur, with initial HFC-induced alterations in for example Glut1 reported returning to normal after a period of HFC feeding) and the precise composition of the diet the animals are fed. Similarly, the link between HFC and cognition remains controversial. Kesby and colleagues [190] have reported no changes in spatial cognition in adult nor aged mice fed high-fat content diet despite clear HFC-induced metabolic dysfunction [190]. However, Kanoski et al. (2010), observed short-lived impairments in spatial memory of mice in the first few days from the start of HFC feeding, which did not persist in animals maintained on long-term HFC [191]. Contrarily, other studies have reported impairments in non-spatial reference and working memory occurring 30 days following a HFC paradigm [191], and persistent long-term cognitive dysfunction whilst fed HFC [192,193]. Interestingly, HFC was found to improve cognitive function in a neurodegenerative mouse model, thus highlighting the complex nature of the relationship between diet, metabolism, and cognition. For an illustrative depiction of the link between age-related cerebrovascular dysfunction associated with neuroinflammation and hypoperfusion, HFC-induced BBB leakage contributing to downregulation of glucose uptake, and the deleterious implications for mitochondrial health leading to cognitive impairment, see Figure 3.

### 5.2. Breakdown of Mitochondrial Function in Age-Related Cognitive Decline.

Hypometabolism in age-related cognitive decline has been linked to the breakdown of mitochondrial bioenergetics, which is characterised by decreased efficiency of oxidative respiration resulting in diminished ATP synthesis, reduced expression of mitochondrial ‘energy genes’ involved in oxidative respiration, and dysregulation of mitochondrial biogenesis. In order to explore the mechanisms underlying age-related mitochondrial impairments, animal models such as the senescence accelerated mice (SAM) strains have been developed [194,195]. Mitochondrial glucose metabolism in the brain of SAMP8 mice (accelerated senescence prone 8), a naturally occurring mouse line that displays a phenotype of accelerated aging, characterised by a memory and learning impairment phenotype, was found to diminish with age as assessed by a decrease in incorporation of carbon 13 (C13) [196]. A longitudinal study in NMRI mice, which tracked the cognitive performance and efficiency of energy metabolism throughout the lifespan (3–24 months), found reduced ATP levels in dissociated brain cells and reduced respiration of complexes I and IV in aged mice compared to young controls [197]. Similarly, brains of aged rhesus monkeys were marked by a significant reduction in the activity of mitochondrial complexes I and IV [198]. Analysis of mitochondrial function in aging using directly reprogrammed neurons (iN) from elderly donors demonstrated a reduction in energy production and significant downregulation in 70% of all mitochondrial genes compared to iN from young donors, with genes involved in electron transport chain complexes I, III, IV, and V most severely affected [199]. Reduced expression of mitochondrial phosphorylation genes was accompanied by a reduction in abundance of the proteins they encode. Interestingly, no changes in mitochondrial biogenesis were reported in the old iN model [199], whereas in the aged Naval Medical Research Institute (NMRI) mice [197] the expression of mitochondrial mass marker citrate synthase was reduced at 18 months. Notably, genes encoding antioxidant defence enzymes superoxide dismutase 2 (SOD2) and catalase (Cat) were shown to progressively decline starting from 18 months of age in those animals. Similarly, senescence accelerated mice P8 (SAMP8) mice, when compared to age-matched accelerated senescence-resistant 1 (SAMR1) mice, show reductions in superoxide dismutase (SOD) activity (Manganese SOD, MnSOD, and copper-zinc SOD, Cu/Zn-SOD) and catalase activity, as well as reductions in levels of GSH—all three well known antioxidant defence mechanisms [200,201]. Perturbations of these antioxidant systems resulted in disruption of OXPHOS via reductions in complex I, complex IV, and ATP synthase activity, ultimately abrogating ATP production [201,202]. More recently, Wang and colleagues showed similar results in SAMP10 mice (a sub-strain of SAM) with onset of pathology at 8-months characterised by decreased SOD activity as well as an increase in lipid peroxidation [203]. Together, these may contribute to the appearance of age-related impairments in learning and memory.

### 5.3. Diet Contribution to Synaptic Dysfunction in Aging

Aging has been shown to lead to cognitive impairment and dementia. Simultaneously, there is also an increased older population that does not adopt healthy lifestyles [204,205]. In this regard, several studies have linked nutrition and aging to inflammation, mitochondrial dysfunction, and ROS build up in the brain [206,207]. Indeed a synergistic role of age and Western diets (WD) has been pointed out [208]. It has been reported that consumption of WD in rats affects synaptic plasticity, thus leading to altered neuronal morphology, dendritic integrity, and blood vessel structure in the hippocampus [209]. Therefore, dendritic and synaptic plasticity changes [210,211], together with changes in the levels of neurotransmitters (dopamine, norepinephrine, serotonin, γ-aminobutyric acid, acetylcholine) and brain-derived neurotrophic factor (BDNF) in the aging brain have been reported, among others [212,213,214,215,216,217].

Synaptic plasticity relies on the enzymatic activity of metalloproteinase-9 (MMP-9) [218,219] and BDNF secretion [220,221]. The upregulation of MMP-9 has been suggested to be involved in several neurodegenerative disorders [222], since higher MMP-9 activity and concentration are related to cognitive impairment [223,224,225]. Additionally, it also plays a role in inflammation [226], which we have presented as one of main causes of mitochondrial dysfunction in aging. Moreover, peripheral BDNF concentration has been reduced with aging [227], and due to its ability to cross the BBB, it has also been linked to cognitive dysfunction in an age-dependent manner.

MMP-9 and BDNF have also been shown to be decreased in an age-dependent manner which is exacerbated by WD [208]. In light of these results, a recent study from Uba Chupel et al. (2021) [228] examined the impact of taurine supplementation on these markers and reported a reduction of MMP-9 levels, which are increased in senescence [229], suggesting a shift in inflammatory balance since taurine supplementation reduces the interleukin 1-beta (IL-1β)/interleukin-1 receptor antagonist (IL-1ra) ratio in elderly women [230] and in vitro studies [231]. However, the authors did not find upregulation of BDNF expression with taurine supplementation as it was shown in different animal models [232]. These results could be pointing out that taurine supplementation, which acts as an inhibitory neurotransmitter [233], mitigates neuroinflammation, mitochondrial dysfunction, and enhances synaptic function, may benefit cognition [234] in aging, especially when subjected to unhealthy nutritional conditions.

Another example of the relevance of the diet when improving synaptic dysfunction in aging could be illustrated through the availability of docosahexaenoic acid (DHA), an n-3 highly unsaturated fatty acid enriched in the central nervous system, which is important for normal brain development. DHA has been shown to promote neurite outgrowth and synaptogenesis [235]. Moreover, in DHA-deficient mouse brains, significant alterations of the synaptic proteome have been identified [236]. Sidhu et al. (2017) cemented the role of DHA in synaptic function by demonstrating downregulation of synaptic proteins during aging which was dependent on the dietary intake of DHA. Consequently, a 24 week dietary supplement of 900 mg/day DHA, as shown by Yurko-Mauro et al. (2010) [237], was sufficient in improving learning and memory in age-related cognitive decline of healthy individuals. These results are providing evidence of the impact of nutrition on synaptic integrity and cognition in aging.

## 6. The Role of Diet in Aging

As we have previously suggested, the impact of nutrition on cognition has been broadly documented in humans. Starting from studies on cognitive development, it has been reported how nutritional intake, independent of social factors, can affect cognitive development. For instance, early evidence showed that a high-protein calorie intake in children’s diets (and of their mothers during lactation), is associated with a higher probability to score better at cognitive tests [238]. Conversely, a relationship between protein-energy malnutrition (defined by height for age) and cognitive development was found in children of developed countries [239]. Although an evident limitation of research on the effects of nutrition and cognitive performance resides in the paucity of longitudinal studies [240], in general terms, it is commonly acknowledged that nutrition (and malnutrition) has a heavy impact on brain development [241,242,243]. However, the importance of getting the right nutrients extends beyond early developmental stages. Increasing evidence associates older adults’ brain health to certain “prudent” dietary patterns, which include a high intake of antioxidants, essential nutrients, and other food-derived bioactive compounds such as polyphenols, through a substantial consumption of fish, fruit, vegetables, low fat dairy, and beverages such as wine, coffee, and tea [15,244,245]. Among the many dietary patterns which can be found around the globe, the Mediterranean diet (MeDi) is certainly one which has been identified to bring about great health benefits. Starting with the notorious Seven Countries Study [246], MeDi has received a significant amount of attention for its role in preserving cardiovascular health and cognitive health too. A meta-analysis on more than 34,000 participants, with a baseline age of 45 or above, showed that the highest adherence to MeDi was inversely linked to the development of cognitive disorders and found an approximately linear relationship between the incident risk of cognitive disorders and general adherence to MeDi [247]. An inverse relationship between adherence to the MeDi and dementia was found in a sample of 1865 Greek elders, where the adherence to the MeDi was also associated with better performance in memory, language, visuospatial perception, and the composite cognitive score; importantly, the strongest association found was for memory [248].

Among the various compounds sourced by MeDi, particular relevant to brain health are the essential long chain omega-3 polyunsaturated fatty acid (LCn-3PUFA), as well as Eicosapentaenoic acid (EPA) and Docosahexaenoic acid (DHA), which are not synthesized in the body, but are present in microalgae or fatty fish [249,250]. In a recent review of cross-sectional and longitudinal studies with healthy older adults, higher omega-3 blood levels were consistently found to be associated with higher hippocampal volume [251]. Some of the reviewed studies also reported a larger total grey matter, total brain volume, and lower white matter lesion volume in association with higher omega-3 [251]. A 6-month double-blind, randomised controlled trial (RTC) on fifty people aged >65 years with mild cognitive impairment found that a diet supplemented with DHA increased verbal fluency [252], and in a recent review on the effects of LCn-3PUFAs supplementation on older adults’ cognition, ten out of the fourteen RCTs reviewed were found to report a positive outcome on at least one domain of cognitive function (working memory, executive function, verbal memory, short-term memory, perceptual speed, etc.) [253]. The efficacy of omega-3 fatty acids encompasses psychiatric disorders as well. Beneficial effects were found in treating depressive symptoms in patients with major depression and, to a lesser degree, bipolar disorder, with some efficacy also observed in early phases of schizophrenia [252,254]. 

### Diet Interventions Targetting Aging-Mitochondrial Dysfunction

However, LCn-3PUFAs are not the only compounds which are beneficial to brain health. Bioactive compounds like curcumin, astaxanthin, resveratrol, hydroxytyrosol, oleuropein, and spermidine, present both in the Mediterranean and Okinawan diet, have been found to exert their protective functions, enhancing the degradation of damaged mitochondria (mitophagy) via the upregulation of mitophagy mediators, and promote the generation of new mitochondria [255]. Given the role of oxidative stress in driving mitochondrial dysfunction and decline in mitophagy, the antioxidative properties of these compounds would protect against premature brain ageing and neurodegenerative diseases like Alzheimer’s disease (AD) [256]. After all, dysfunction in mitophagy may trigger or worsen neurodegenerative diseases such as AD, where mitochondrial dysfunction plays a central role in the pathogenesis [257,258].

The brain is a highly metabolically active tissue that relies on oxidative phosphorylation as a way for maintaining energy. However, as we have previously described, such mitochondrial processes lead to the production of reactive oxygen species (ROS) that generate oxidative damage. Beyond the initial damage inflicted by oxidative stress, more deleterious effects are achieved through concomitant mitochondrial dysfunction and, acting in unison, elicit alterations in cellular signalling and gene expression as well as modulating cell death pathways, increasing the likelihood of apoptotic events and cellular death, and reducing cellular energy availability [259]. The brain consumes a large amount of oxygen and is highly rich in lipids which are more prone to attack from free radicals and undergo lipid peroxidation, thus becoming prone to oxidative stress [260]. Damage accumulation by oxidative processes is a key mechanism of the ageing progression and a common feature of ageing brains [15,261]. Studies of human autopsy tissue show higher levels of oxidative damage to DNA/RNA, proteins, and lipids in aged as compared to young human brains [262,263]. In the human brain, both pathological and normal ageing see mitochondrial activity to be compromised [264]. Indeed, in normal aging, a series of functions like mitochondrial respiration and metabolism decrease, while the rate of somatic mitochondrial DNA mutations increases [261]. Therefore, antioxidant supplementation therapy could be considered as a means of prevention for normally aged subjects, or as a possible treatment for patients with neurodegenerative disorders like Alzheimer’s and Parkinson’s disease, where ROS and mitochondrial dysfunctions play critical roles [259,265]. However, despite the convincingly positive outcomes in studies using animal models [266,267,268], cross-sectional and case–control studies on human cognition have reported mixed findings, with either a positive, inconclusive, or no significant effects of antioxidant supplementation. For instance, functional status and cognitive functions did not differ in a group of elderly patients according to their vitamin B1 status [269]. Yet, vitamin B1-deficient patients exhibited a higher proportion of Alzheimer’s disease (AD), depression, cardiac failure, and falls [269]. Daily supplementation with high doses of oral vitamin B12 led to a significantly smaller improvement in memory function compared with a placebo group, in a sample of people aged 70 or over [270]. Similarly, vitamin B12 supplementation did not prevent cognitive decline in a group of older (70 or older) diabetic patients with borderline vitamin B12 status [271]. Moore et al. [272] investigated the association between vitamin B12 and cognitive impairment or dementia illustrating that efficacy was only achievable, as measured by cognitive improvement, in patients with pre-existing deficiencies in vitamin B12. Nevertheless, low serum vitamin B12 levels were associated with neurodegenerative disease and cognitive impairment, and small subsets of dementia receive a therapeutic outcome when placed on vitamin B12 supplements [272]. Additionally, low plasma levels of vitamin C are associated with cognitive decline in elderly people [273], and several randomized controlled trials show the beneficial effect of vitamin E supplementation in delaying the functional decline observed during AD progression [274]. Nevertheless, in a longitudinal study with individuals aged 65–105 years, supplement use of vitamins C and/or E did not delay the incidence of dementia or AD [275]. In accordance with this, a recent review including 28 studies with more than 83,000 participants, did not find evidence that any vitamin or mineral supplementation had a meaningful effect on cognitive decline or dementia of healthy adults in mid or late life. Furthermore, the only positive outcome that was noted in long-term studies was supplementation with antioxidant vitamins [276]. Yet, the same authors acknowledged that the studies included in their review tended to be too short to assess the maintenance of cognitive function, that longer studies often had other primary outcomes, and that the cognitive measures used may have lacked sensitivity [276]. Additionally, it should be considered that the therapeutic use of most of these compounds is limited, since the blood–brain barrier reduces the permeability of most antioxidants [277]. For a more extensive review on the relationship between vitamins and dementia, we recommend the recent work by Alam [268].

Along with MeDI, other alternative diet-based interventions like Ketogenic Diet (KD), caloric restriction (60% of the food intake), Dietary Approaches to Stop Hypertension (DASH), and Mediterranean-DASH diet Intervention for Neurological Delay (MIND), have been proven effective to protect against oxidative damage and possibly reduce the pathophysiological hallmarks of neurodegenerative diseases like AD [278]. In particular, in the last decade KD has received a fair amount of attention from human research despite the neuroprotective role of ketosis being known since the 1920s, when physicians introduced it to treat epilepsy [279]. KD is based on the production of ketone bodies by the liver from fatty acids and it can be triggered by fasting or with a diet high in fats and low poor in carbohydrates. In a state of ketosis, the body swaps from glucose to fat as its primary source of fuel [280]. Compared to glycolysis, the metabolism of ketone bodies has a neuroprotective action, having been associated with a lower production of ROS and therefore lower oxidative stress [281]. In particular, the ketone body beta-hydroxybutyrate reduces mitochondrial ROS production and inhibits histone deacetylases, upregulating the transcription of some genes that are protective against oxidative stress [282]. Moreover, ketone bodies contribute to the reduction of ROS production through the expression of mitochondrial uncoupling protein (UCP), thereby decreasing mitochondrial membrane potential [283].

The beneficial effects of KD on human cognitive function have been reported in several studies. One method to mimic KD by enhancing ketone production is to administer large amounts of medium-chain triglycerides (MCT) as part of the diet [284]. A RTC of supplemental MCT showed an improvement in cognitive testing after 45 days, and up to 90 days in those subjects who did not carry the apolipoprotein E4 (ApoE4) allele [285]. Another study directly administered ketone bodies to 20 subjects and recorded improvement on the Alzheimer’s Disease Assessment Scale-Cognitive Subscale (ADAS-cog) on subjects who did not carry the ApoE4 allele A, while higher ketone values were associated with greater memory improvement across all subjects [286]. A clinical intervention in 22 Japanese patients with sporadic AD did not report any improvement in cognitive functioning following ketone body supplementation, even in those patients without the ApoE4 allele. However, some ApoE4-negative patients did show some improvement in their cognitive functions [287]. Thus, some of these studies suggest that neuropsychological effects would depend on the presence of ApoE4 genotype, which is the most prevalent genetic risk factor for AD. Finally, a 6-week randomized cross-over study on 20 older adults with or without a mild cognitive impairment (MCI) showed that a modified MeDi-KD can improve AD biomarkers [288]. However, no differences were observed on memory performance when compared to a control diet [288]. More human studies are needed to disentangle the contribution of the different factors and to understand clearly what could be the potential modulators of this promising intervention.

## 7. Future Directions

Aging is characterized by cognitive impairments where mitochondrial dysfunction has been proved to play a central role. It is well established that a healthy diet reduces the risk of developing cognitive decline in elderly individuals pointing out its potential as a therapeutic. In this regard, antioxidant intake and diet supplementation have been linked to improved cognitive functioning. Antioxidants, such as Ginkgo biloba extract (EGb 761), known to have antioxidant properties, has been proved to decreased oxidative DNA damages and peroxide generation, parallel to an increase in mitochondrial membrane potential in the brain of old (27 months) when compared to young (4 months) animals [289]. Furthermore, in vitro treatment of Drp1 KO cells with antioxidants such as N-acetylcysteine and MitoQ has been shown to reduce mitochondrial swelling and cell death [290]. These results not only point out the role of oxidative stress in age-related mitochondrial impairments, but also the importance of dietetic supplements while aging. Despite these results, we would like to note that the effectiveness of antioxidant supplementation in the diets of elderly individuals could be limited due to the inability of natural antioxidants to reach the ROS producing organelles in the brain. 

We also want to highlight the benefits of long-term fruit supplementation in aging degenerative processes. In this regard, a study from Braidy et al. (2016) [291] has proved that pomegranate diet administered for 15 months enhanced synaptic plasticity, inhibited neuroinflammation and promoted autophagy. All these changes are due to the active components contained in the pomegranate, such as significant amounts of ellagitannin, such as punicalin and punicalagin, and flavonoids, such as kaempferol and quercetin derivatives. Indeed, quercetin became relevant when it was proved that it can modulate pathways associated with mitochondrial biogenesis, mitochondrial membrane potential, oxidative respiration and ATP anabolism, intra-mitochondrial redox status, and subsequently, mitochondria-induced apoptosis [292], thus improving the mitochondrial dysfunction associated to aging. These results pointed out the relevance of the active phytochemicals present in pomegranate extracts to improve brain function and mitigate neurodegeneration in aging.

Moreover, as previously detailed, the relevance of dietary effects on cognitive improvement has been proved by the development of the Mediterranean-DASH Intervention for Neurodegenerative Delay diet (MIND diet), with a focus of improving cognition in dementia sufferers as well as preventing normal age-related cognitive decline in the elderly [293]. Both the MIND and MeDi diet utilise plant-based foods with consumption of red meats and saturated fats limited. However, the MIND diet pays special attention to specific berries and leafy-green vegetables, which are seen to be cognition enhancers, such as spinach and blueberries. Morris et al. (2014) showed that MIND diet was associated with a slower rate of cognitive decline, however, more needs to be investigated regarding the mechanisms behind this improvement. Indeed, aside from its antioxidant effects, changes in diet may induce epigenetic and neurogenic changes [294] in elderly that should be also explored.

Rodent models have highlighted the importance of antioxidants and exercise in improving cognitive function by demonstrating that enriched diets and exercise can reduce ROS production, enhance mitochondrial function, thereby improving bioenergetic dynamics of the cell, and preserve synaptic activity to enable functioning neuronal networks. Furthermore, the use of antioxidants in the diets of elderly individuals has shown beneficial effects, shedding light on the need for further investigation. This review critically assessed the current status of healthy diets on recovering mitochondrial dysfunction in elderly individuals. We also want to highlight that healthy diets should be accompanied by regular exercise and a better lifestyle, which are likely to delay the progression of dementia and mitochondrial dysfunction in elderly individuals. Finally, we believe that new therapeutic approaches should factor in age and unhealthy diets when assessing the efficacy of their treatments, in order to better translate experimental findings into clinically-relevant interventions.
